# Effects of Vitamin D Supplementation and Baseline Vitamin D Status on Acute Respiratory Infections and Cathelicidin: A Randomized Controlled Trial

**DOI:** 10.1093/ofid/ofae482

**Published:** 2024-08-27

**Authors:** Akseli Laaksi, Heikki Kyröläinen, Harri Pihlajamäki, Jani P Vaara, Tiina Luukkaala, Ilkka Laaksi

**Affiliations:** Faculty of Medicine and Health Technology, University of Tampere, Tampere, Finland; Department of Leadership and Military Pedagogy, National Defence University, Helsinki, Finland; Faculty of Sport and Health Sciences, University of Jyväskylä, Jyväskylä, Finland; Faculty of Medicine and Health Technology, University of Tampere, Tampere, Finland; Department of Orthopedics and Traumatology, Seinäjoki Central Hospital, Seinäjoki, Finland; Department of Leadership and Military Pedagogy, National Defence University, Helsinki, Finland; Research, Development and Innovation Center, Tampere University Hospital, Tampere, Finland; Health Sciences, Faculty of Social Sciences, University of Tampere, Tampere, Finland; Faculty of Medicine and Health Technology, University of Tampere, Tampere, Finland; Centre for Military Medicine, Finnish Defence Forces, Riihimäki, Finland

**Keywords:** acute respiratory infection, cathelicidin, randomized controlled trial, vitamin d, young men

## Abstract

**Background:**

Vitamin D supplementation may lower the risk of acute respiratory infection (ARI), and the effects may be mediated through the induction of cathelicidin production.

**Objective:**

To study the effect of vitamin D supplementation on ARI and cathelicidin concentration in a randomized controlled trial (RCT) and to study the associations between baseline serum 25 hydroxyvitamin D (25(OH)D) and ARIs and cathelicidin concentrations in a 14-week follow-up study.

**Methods:**

In the RCT study, the participants were randomized into 2 groups to receive either 20 µg of vitamin D_3_ or an identical placebo daily. Blood samples were obtained 3 times, at the beginning (study week 0), mid-term (study week 6), and at the end of the study period (study week 14). The follow-up study had 412 voluntary young men from 2 different locations and seasons (January and July). The primary outcomes were the number of ARIs diagnosed and the number of days off because of ARI.

**Results:**

In the RCT, vitamin D supplementation had no effect on ARI or days off because of ARI. However, regardless of the group, vitamin D insufficiency (<50 nmol/L) was associated with increased ARI. In the 14-week follow-up study, insufficient serum 25(OH)D at baseline was also associated with increased risk of ARI (odds ratio [OR], 2.1; 95% confidence interval [CI], 1.2–3.7) and also days-off duty (OR, 2.3; 95% CI, 1.3–4.0) and was inversely associated with cathelicidin concentration (OR, 0.49; 95% CI, .24–.99).

**Conclusions:**

Sufficient serum 25(OH)D may be preventive against acute respiratory infection and the preventive effect could be mediated through the induction of cathelicidin production.

***Clinical Trial Registry number:*** NCT05014048. https://clinicaltrials.gov/study/NCT05014048?term=NCT05014048&rank=1

Acute respiratory infection (ARI) is 1 of the most common diseases in the world. In the United States, the average adult suffers from 2 to 4 infections annually [1]. Particularly since the COVID-19 pandemic, there has been growing interest in potential preventing factors, 1 of which is vitamin D. The possible preventive effect of vitamin D is supported by laboratory findings that show vitamin D acting as part of innate immune responses, with the important component being the induction of human cathelicidin, or in short cathelicidin. Cathelicidin is an antimicrobial protein that protects from respiratory infections as a part of the first line defense of human immunity [2–4]. The results of observational studies investigating low serum 25 hydroxyvitamin D (25(OH)D) as a risk factor for ARI have been consistent [5, 6]. As for randomized controlled trials (RCTs), the results vary [7]. A recent meta-analysis including 43 RCTs found that the preventive effect of vitamin D depends on frequency, dose, and duration of the supplementation. The study showed that daily administration of 10–25 µg of vitamin D supplementation for 12 months or less reduced the risk of respiratory infections by 30% [7].

Thus, the main purpose of this study was to study the effect of daily administered 20 µg vitamin D_3_ supplementation on ARIs, days off because of ARIs, and cathelicidin concentrations during military service in young adult men through a double-blinded, placebo-controlled RCT. The trial lasted 14 weeks, from January through May. The secondary aim was to investigate the relationship between baseline serum 25(OH)D and previously mentioned variables in a large (n = 412) 14-week follow-up study.

## METHODS

### Study Protocol

The study had 2 approaches: First, a double-blinded, placebo-controlled RCT lasting from January through May. The trial was conducted during the years 2018–2019. In the RCT study, the participants were randomized into 2 groups using a computerized procedure, supplementation (n = 76), and a control group (n = 88). The supplementation group received 20 µg (800 IU) of vitamin D_3_ daily (intention-to-treat), whereas the control group received a placebo. The supplemented dose was chosen based on a recent meta-analysis at the time of the initiation of the trial [8]. Second, a follow-up study with 412 voluntary conscripts from 2 different locations and seasons (January and July). For the follow-up study, the baseline blood samples of the participants were drawn either in January (n = 184) or July (n = 228). The sufficient limit of 50 nmol/L set by The Nordic Nutrition Recommendations was used in the study [9].

The medical records of all participants for the study period of 14 weeks were reviewed. The primary outcomes collected were the amount of ARIs diagnosed by a healthcare professional, and the number of days off duty because of ARIs.

Season, body mass index (BMI), and aerobic fitness were used as covariates. They were selected for their possible confounding effects: season for its effects on serum 25(OH)D through differences in sunlight exposure, BMI for its possible association with low serum 25(OH)D [10], and aerobic fitness, measured by a 12-minute running test [11], for its possible effects on serum 25(OH)D [12]. In the follow-up study, the use of vitamin D supplements was additionally used as a covariate.

### Intervention and Randomization

Participants were randomized in a 1:1 ratio to receive a 20 µg vitamin D_3_ supplementation or an identical placebo. The baseline characteristics (height, weight, BMI, aerobic fitness, serum 25(OH)D, cathelicidin concentration) (Table 1**)** of the participants between groups did not differ. Throughout the study period, the conditions regarding physical activity, nutrition, clothing, accommodation, and exposure to sunlight were homogenous between groups. The final group sizes due to dropouts were 113 in group 1 and 118 in group 2.

**Table 1.** Differences Between Groups Were Tested Using the Mann-Whitney *U* test

	Placebo (N = 113)			Supplementation (N = 118)			1 Versus 2
	n	Md	(IQR, Range)	n	Md	(IQR, Range)	*P*
Height (cm)	92	177	(174–183; 160–196)	85	180	(176–184; 166–193)	.06
Body mass (kg)	91	74	(69–82; 51–142)	85	76	(69–85; 58–109)	.43
BMI	91	24	(22–26; 18–41)	85	24	(21–26; 17–33)	.97
Aerobic fitness (km)	93	2.5	(2.2–2.6; 1.2–3.0)	83	2.5	(2.3–2.6; 0.6–3.0)	.79
S-25(OH)D (nmol/L)	96	55	(45–66; 24–123)	88	55	(47–65; 18–89)	.57
Cathelicidin (ng/ml)	96	38.7	(31.0–50.0; 12–731)	88	40.0	(32.9–58.5;19–743)	.29

Abbreviations: BMI, body mass index; IQR, interquartile range; Md, median; S-25(OH)D, Serum 25(OH)D.

The intervention was implemented as an intention-to-treat protocol. The participants received the supplement or placebo tablets at the beginning of the study and were instructed to take 1 tablet daily. The containers for the supplements or placebos were uniquely identified with either the number 1 or 2 for distinction. Participants, healthcare providers, investigators, and outcome assessors were all blinded from the intervention during the study. After the data were analyzed, a sealed envelope was opened to reveal the groups.

### Participants

A total of 184 (age 19 ± 1 year, height 179 ± 7 cm, body mass 78 ± 12 kg, BMI 24 ± 3) voluntary conscripts were included in the RCT and 412 (age 19 ± 1 year, height 179 ± 7 cm, body mass 75 ± 13 kg, BMI 24 ± 4) were included in the follow-up. The inclusion criteria was passing the entry medical examination as healthy, the age of 18–29 years. Also, only male participants were included because of the limited number of female conscripts.

### Patient Consent Statement

An information session of the study was organized for the participants, after which they also received a written information. During and after the event, participants were able to ask questions and get more information. After the session, written informed consent was requested from the participants. Participation in the study was completely voluntary, and the participant could withdraw from the study at any time without giving a reason. Research data that had already been collected were not removed from the research material unless the participant specifically requested it. Special attention was paid to the implementation of volunteering because conscripts constitute a unique research population. The study was approved by the Ethics Committee of the Pirkanmaa Hospital District, ETL code R17155, HUS/1557/2018, and the Finnish Defence Forces (AN21508, AP10027, AQ24718, AR13336). The study was registered in the Clinical Trials Registry (NCT05014048) according to the criteria of the International Committee of Medical Journal Editors (ICMJE).

### Blood Samples and Acute Respiratory Infection Data

Blood samples were obtained three times, at the beginning (study week 0), mid-term (study week 6), and at the end of the study period (study week 14). The vitamin D status was evaluated by widely accepted biomarker 25(OH)D [13]. The measurements of serum 25(OH)D were performed using electrochemiluminescence immunoassay. Vitamin D can be obtained in 2 different forms: D_3_, which is produced in the skin of animals, and D_2_, which is plant-based vitamin D [14]. The method that was used in the present study shows the combined concentration of the D_3_ and D_2_ forms. The electrochemiluminescence immunoassay is in continuous use in large good laboratory practice evaluated and authorized Finnish central laboratory continuously analyzing clinical 25(OH)D patient samples. The device is regularly tested by a set of calibration standards with known concentrations of 25(OH)D. The measurements were evaluated in the accredited laboratory of the Clinical Chemistry and Medical Research Unit of Seinäjoki Central Hospital. The measurements of human cathelicidin were evaluated using the Hycult human LL-37 ELISA kit (Uden, Netherlands) at the University of Jyväskylä. The inter-assay coefficient of variance was 13% and the sensitivity was 1.7 ng/mL.

The medical records for all participants covering 14 weeks of the study were reviewed, and any diagnosed acute respiratory tract infection (sinusitis, tonsillitis, bronchitis, otitis, pneumonia, laryngitis, and pharyngitis) was recorded. The inclusion criteria was that the ARI led to days off duty and was diagnosed by a healthcare professional.

### Statistical Methods

The sample size calculations were made on the assumption that, during the study period, conscripts would have an average of 6 (standard deviation [SD] 2–4) days off because of ARI. A daily vitamin D supplement of 20 µg was assumed to reduce the number of days off because of ARI by 20% compared to the placebo group [8]. Sample size requirement was 47/group (SD 2), 104/group (SD 3), and 184/group (SD 4). The sample size was calculated with a power assumption of 80% (alpha 5%, G*Power 3.1.9.2, Wilcoxon-Mann-Whitney).

BMI, aerobic fitness, serum 25(OH)D, cathelicidin, ARI, and days off because of the ARI were categorized according to the lower or upper interquartile range. Differences between control group (placebo) and supplementation group (daily 20 µg vitamin D supplement) were analyzed using the Fisher-Freeman-Halton test. Baseline characteristic differences between groups were tested using Mann-Whitney *U* test.

Upper quartile of ARI, days off because of the ARI, and cathelicidin were modeled using logistic regression analysis. Interaction effects between serum 25(OH)D (<50 vs ≥ 50 nmol/L) and use of vitamin D supplementation (yes vs no) and season (winter vs summer) were tested. Statistically significant (*P* < .05) interactions with 25(OH)D were included in multivariable-adjusted model. BMI (≥25 vs < 25) and aerobic fitness (<2500 vs ≥ 2500) were included as adjusting factors in the models. Results were shown by odds ratios with 95% confidence intervals.

Adjusted effect of serum 25(OH)D on cathelicidin, ARI, and days off because of the ARI were modelled as binomial, distributed over weeks (0, 6, 14) using Generalized Linear Mixed Models. Participants created potential sources of variation, and therefore this subject-specific effect was included as random effect in the model [15]. Explanatory factors were modelled as fixed effects. The Generalized Linear Mixed Models was applied with the R statistical software package (version 4.0.3, lme4 function, R Core Team (2018); R: A language and environment for statistical computing, R Foundation for Statistical Computing, Vienna, Austria; https://www.R-project.org/). Other statistical analyses were performed using SPSS for Windows (IBM Corp. Released 2021. IBM SPSS Statistics for Windows, Version 28.0. Armonk, NY: IBM Corp). Two-sided *P* values < .05 were considered statistically significant.

## RESULTS

### RCT Study

**Figure 1.**
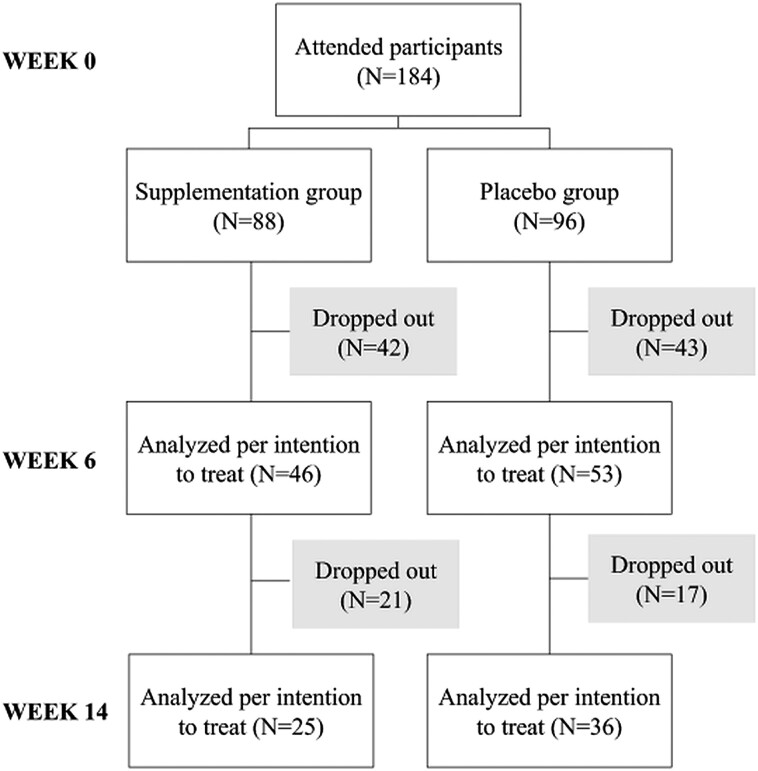
Participant flow-chart of the randomized controlled trial study.

As shown in Figure 1, a total of 184 participants attended the RCT. At the beginning of the study, there were no statistically significant differences in characteristics between the supplementation and the placebo groups (Table 1). A total of 123 participants dropped out during 14-week follow-up.

The group had no effect on ARIs, days off because of ARIs, or cathelicidin concentrations. However, those with insufficient serum 25(OH)D had more ARIs in the whole-study population (Table 2).

**Table 2.** The Effect of Vitamin D Supplementation on Acute Respiratory Infections (ARI), Days off Due to the ARI and Cathelicidin Concentrations

	ARI ≥ 2 Versus 0–1		Days Off Because of The ARI ≥ 3 Versus 0–2 D		Cathelicidin ≥ 55 Versus < 55 ng/mL	
	OR 95% Cl	*P*	OR 95% Cl	*P*	OR 95% Cl	
Supplement						
						
Placebo	1.00		1.00		1.00	
Vitamin D	1.01 (.96–1.05)	.79	1.04 (.97–1.12)	.29	1.06 (.94–1.20)	.35
25(OH)D, nmol/L						
						
<50	1.00		1.000		1.000	
≥50	.93 (.89–.98)	.003	.99 (.93–1.07)	.99	1.04 (.93–1.17)	.50
Wk						
						
0	.92 (.87–.98)	.008	.76 (.70–.83)	<.0001	1.00	
6	1.03 (.96–1.10)	.43	1.07 (.97–1.18)	.20	1.09 (.95–1.25)	.21
14	1.00		1.00		1.22 (1.04–1.42)	.01
BMI^a^						
						
≥25	1.00		1.00		1.00	
<25	.99 (.94–1.05)	.80	1.01 (.93–1.10)	.90	.98 (.85–1.13)	.72
Aerobic fitness^a^						
<2500 m	1.00		1.000		1.000	
≥2500 m	.98 (.93–1.02)	.29	.93 (.86–1.00)	.05	.87 (.76–.97)	.02

ARI and days off were modelled as binomial distributed over weeks (0, 6, 14) using generalized linear mixed models (GLMM) among 184 conscripts with 339 observations. Interactions with 25(OH)D were statistically nonsignificant.

Abbreviations: 25(OH)D, 25 hydroxyvitamin D; ARI, acute respiratory infection; BMI, body mass index; CI, confidence interval; OR, odds ratio.

^a^Results for unknowns group are not shown.

**Figure 2.**
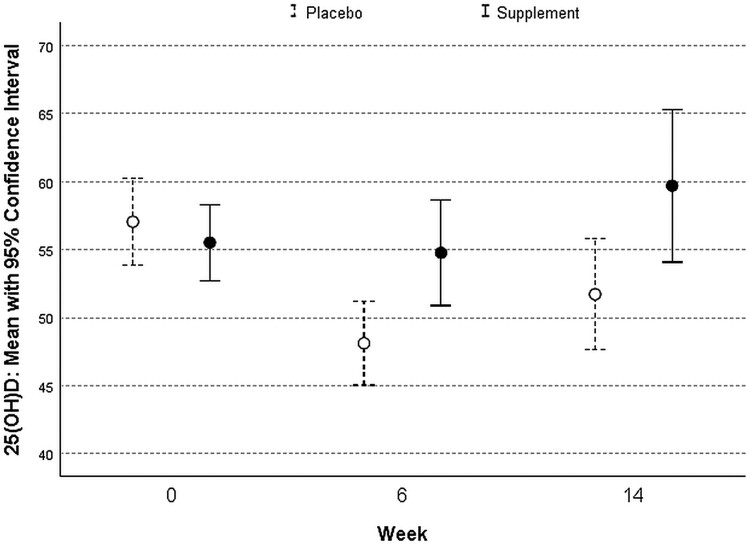
The variation of serum 25(OH)D between groups during the 14-wk randomized controlled trial study.

The mean serum 25(OH)D in the beginning of the study in placebo group was 57.0 ± 15.6 nmol/L and 55.5 ± 13.2 nmol/L in the supplementation group, respectively. In study week 6, the mean vitamin D level was 48.1 ± 11.2 nmol/L in placebo group and 54.8 ± 13.0 nmol/L in the supplement group (Figure 2). In study week 14, the mean vitamin D level was 51.7 ± 12.1 in placebo group and 59.7 ± 13.6 in the supplement group. The tested differences between study groups were statistically significant (*P* = .007 for 6-week and *P* = .019 for 14-week timepoint). Still, because of the overlap of confidence intervals, the difference between groups may be only near the statistical significance.

### Follow-up Study

The mean serum 25(OH)D was 68.5 ± 20.0 nmol/L for the whole-study population in the beginning of the study (study week 0, January, and July). The participants that started the trial in January (56.3 ± 14.5 nmol/L) had significantly (*P* < .001) lower serum 25(OH)D levels than the ones that started in July (78.3 ± 18.5 nmol/L). In the winter population, 33.7% were vitamin D insufficient (<50 nmol/L), whereas in the summer population the respective percentage was only 5.3%.

Multivariable-adjusted insufficient serum 25(OH)D at baseline was associated with a higher incidence of ARI and days off because of ARI and lower concentration of cathelicidin (Table 3).

**Table 3.** The Associations of Baseline Characteristics on ARI, Days off because of ARI, and Cathelicidine Through 14-wk Follow-up

	ARI (N = 403)	D Off Because of ARI (N = 399)	Cathelicidin (N = 401)
	≥2 versus 0–1	≥6 versus 0–5	≥55 versus < 55
	OR (95% CI)	OR (95% CI)	OR (95% CI)
S-25(OH)D (nmol/L)			
≥50	1.00	1.00	1.00
<50	2.11 (1.19–3.74)	2.26 (1.27–4.03)	.49 (.24–.99)
Season			
Summer	1.00	1.00	1.00
Winter	1.95 (1.10–3.45)	1.25 (.70–2.25)	1.06 (.57–1.95)
Use of vitamin D supplement			
Yes	1.00	1.00	1.00
No	1.44 (.76–2.70)	.94 (.49–1.79)	.74 (.37–1.46)
Body mass index			
<25	1.00	1.00	1.00
≥25	.86 (.50–1.48)	.75 (.43–1.30)	1.33 (.77–2.30)
Aerobic fitness (m)			
≥2500	1.00	1.00	1.00
<2500	1.60 (.93–2.75)	1.66 (.96–2.85)	1.30 (.75–2.27)
Interactions			
S-25(OH)D*Group	*P* = .75	*P* = .24	*P* = .45
S-25(OH)D*Season	*P* = .29	*P* = .17	*P* = .78

Multivariable-adjusted logistic regression. Interaction effects between S-25(OH)D with group and season were tested. Statistically nonsignificant interactions *P* ≥ .05 were excluded from the models.

Abbreviations: S-25(OH)D, Serum 25(OH)D; ARI, acute respiratory infection; CI, confidence interval; OR, odds ratio.

## DISCUSSION

The main purpose of the present study was to investigate the effects of vitamin D_3_ supplementation on ARIs and cathelicidin concentrations. Throughout the 14-week RCT study, no statistically significant differences were found between the placebo and supplementation groups. However, regardless of the group, vitamin D insufficiency was associated with an increased risk of ARI. The secondary purpose was to investigate the relationship between baseline serum 25(OH)D and the risk of ARI, days off duty because of ARI, and cathelicidin concentrations in a 14-week follow-up study. Interestingly, the participants with vitamin D insufficiency at baseline had a 2.1-fold risk of having 2 or more ARIs and a 2.3-fold risk of having 6 or more days off duty because of ARI when controlled for possible confounding factors. Additionally, vitamin D associated with higher cathelicidin concentrations.

In the present study, no effect of vitamin D supplementation on ARIs was observed. The observed lack of differences between the groups may be attributed from the loss of power because of participants dropping out. It is noteworthy that the participants with vitamin D insufficiency had a higher risk of ARI across both groups. Although there are a limited number of similar RCTs studying the effect of vitamin D supplementation on ARI or cathelicidin specifically in young healthy men, previous studies have demonstrated promising outcomes. These include a reduction of ARI symptoms [16] and fewer days off because of ARI [17]. Nonetheless, the heterogeneity of the studies' supplemented dosage rises as a problem pursuing to determine the most effective dose for preventing the ARIs. In a 2021 meta-analysis, the preventive effect of vitamin D supplementation was found to depend on the frequency, dosage, and duration of the supplementation. The most beneficial effect was observed in studies in which the supplement was administered daily and the dosage was 10–25 μg (400–1000 IU) lasting up to 12 months [7]. The supplemented amount of vitamin D in the present study was indeed 20 μg (800 IU) administered daily.

Furthermore, the present 14-week follow-up study showed baseline vitamin D insufficiency increasing the risk of ARIs and having more days off duty because of ARIs. In addition, vitamin D associated with higher cathelicidin concentrations. The findings of the present study strengthen the hypothesis of the preventive effect of vitamin D for ARIs as the results coincide with the previous observational studies [5, 6]. The connection between vitamin D and the immune system has been acknowledged for many years and there are several potential pathways [3]. The dietary or endogenously obtained vitamin D undergoes hydroxylation in the liver to be converted into 25(OH)D. The assessment of vitamin D status is conducted through the measurement of serum 25(OH)D level [13]. 25(OH)D is then transported to the kidneys or specific tissues, where it undergoes another round of hydroxylation, transforming into its most active form, 1,25(OH)_2_D [14]. 1,25(OH)_2_D exerts its effects through a cell receptor known as the vitamin D receptor (VDR) [18]. Immune cells have been shown to express the VDR and some can even express the enzyme 1α-hydroxylase, making them capable of converting 25(OH)D into 1,25(OH)_2_D. When a pathogen enters the body, a type of pathogen-recognition receptors, called toll-like receptors activate in monocytes leading to increased transcription of VDR and 1-alfa-hydroxylase-enzyme. Then the circulating 25(OH)D can be converted into 1,25(OH)_2_D within monocytes, which bind onto VDRs and act as a transcription factor for cathelicidin induction. This can also take place in respiratory epithelial cells [2–4]. This is supported by the findings of vitamin D correlating with cathelicidin in the present study. However, there were no significant differences in cathelicidin between groups. The findings of prior research are inconsistent showing results both for [19–21] and against [16, 22] the hypothesis of the present study. There are limited studies on this topic, and more research is needed.

Interestingly, the present study shows a large prevalence of vitamin D insufficiency in participants during winter months. In the baseline measurements, one third of the participants were vitamin D insufficient and by March, more than half of the participants in the placebo group were insufficient. However, in the supplementation group, the majority of the participants were sufficient. Despite the sample being relatively small, the results raise concerns on whether the vitamin D intake is too low in Finland during winter months in young adult men. The results coincide with a recent study that determined vitamin D status of Finnish adolescents [23]. In northern latitude countries, such as Finland, the adequate amount of sunlight exposure for vitamin D production in the skin is only possible between March and October [24]. Outside of that period, the only vitamin D supply comes from the diet. Considering that it is well known that the incidence of viral infections such as common cold and influenza significantly rise during winter months [25], the findings are particularly noteworthy.

The present study has several strengths. Because of military service, the conditions between the groups were homogenous concerning food, physical exertion, accommodation, sleeping hours, clothing, and sunlight exposure. Also, military conditions, where conscripts live close together in shared rooms, are favorable for ARIs. In addition, the study design was based on the recent strong evidence of the dosage, frequency, and duration of the supplementation. However, the present study has some limitations. There were more participants lost to follow-up than expected, which may have affected the results. Also, the intention-to-treat study design does not give certainty whether the participants took the intended doses daily.

In conclusion, the present study shows that there is a possible preventive association between sufficient serum 25(OH)D and ARIs, and the association could be through the induction of cathelicidin production. However, further RCT research is needed to determine the optimal amount of vitamin D supplement for the possible preventive effect. The present study provides valuable information to guide future RCTs to conduct similar studies with possibly higher doses of vitamin D administered daily.

## Notes

***Acknowledgments***. The authors thank Liisa Rantahalvari for her assistance during the data collection. The authors also thank Juha-Petri Ruohola and Jussi Laaksonen for their contribution to launching this study and Onni Niemelä, Susanna Luoma, and Tanja Toivanen for conducting laboratory analysis. The authors also thank Macey Higdon for the revision of the language and Juuso Pöytäkivi and Kim Kotilainen for technical assistance in data analysis.

***Author Contributions***. A.L. and T.L. contributed to the creation and analysis of the data. A.L., I.L., J.V., H.P., and H.K. contributed to the conception and design of the study. A.L. wrote the first draft of the manuscript. All authors contributed to editing and approved the final manuscript. I.L. had primary responsibility for final content.

***Data sharing statement***. Data described in the manuscript, code book, and analytic code will be made available upon request.

***Financial support***. This study was funded by the Centre for Military Medicine Finnish *Defence Forces* , *State Research Financial support* (PSHP), and National Defense Support Foundation (MPKS).

## References

[1] Heikkinen T, Järvinen A. The common cold. Lancet Rheumatol 2003; 361:51–9. 10.1016/S0140-6736(03)12162-9 PMC7112468 12517470

[2] Ahmed A, Siman-Tov G, Hall G, Bhalla N, Narayanan A. Human antimicrobial peptides as therapeutics for viral infections. Viruses 2019; 11:704. 31374901 10.3390/v11080704 PMC6722670

[3] Bishop EL, Ismailova A, Dimeloe S, Hewison M, White JH. Vitamin D and immune regulation: antibacterial, antiviral, anti-inflammatory. JBMR Plus 2021; 5:e10405. 32904944 10.1002/jbm4.10405 PMC7461279

[4] Greiller CL, Martineau AR. Modulation of the immune response to respiratory viruses by vitamin D. Nutrients 2015; 7:4240–70. 26035247 10.3390/nu7064240 PMC4488782

[5] Jolliffe DA, Griffiths CJ, Martineau AR. Vitamin D in the prevention of acute respiratory infection: systematic review of clinical studies. J Steroid Biochem Mol Biol 2013; 136:321–9. 23220552 10.1016/j.jsbmb.2012.11.017

[6] Pham H, Rahman A, Majidi A, Waterhouse M, Neale RE. Acute respiratory tract infection and 25-hydroxyvitamin D concentration: a systematic review and meta-analysis. Int J Environ Res Public Health 2019; 16:3020. 31438516 10.3390/ijerph16173020 PMC6747229

[7] Jolliffe DA, Camargo CA, Sluyter JD, et al Vitamin D supplementation to prevent acute respiratory infections: a systematic review and meta-analysis of aggregate data from randomised controlled trials. Lancet Diabetes Endocrinol 2021; 9:276–92. 33798465 10.1016/S2213-8587(21)00051-6

[8] Martineau AR, Jolliffe DA, Hooper RL, et al Vitamin D supplementation to prevent acute respiratory tract infections: systematic review and meta-analysis of individual participant data. BMJ 2017; 356:i6583. 28202713 10.1136/bmj.i6583 PMC5310969

[9] Nordic Council of Ministers . Integrating nutrition and physical activity. Nordic Nutrition Recommendations 2012. Vol Report No: 5 2014. doi:10.6027/Nord2014-002.

[10] Yao Y, Zhu L, He L, et al A meta-analysis of the relationship between vitamin D deficiency and obesity. Int J Clin Exp Med 2015; 8:14977–84. 26628980 PMC4658869

[11] Cooper KH . A means of assessing maximal oxygen intake: correlation between field and treadmill testing. JAMA 1968; 203:201–4. 5694044

[12] Laaksi I, Ruohola JP, Tuohimaa P, et al An association of serum vitamin D concentrations <40 nmol/L with acute respiratory tract infection in young Finnish men. Am J Clin Nutr 2007; 86:714–7. 17823437 10.1093/ajcn/86.3.714

[13] Utiger RD . The need for more vitamin D. N Engl J Med 1998; 338:828–9. 9504945 10.1056/NEJM199803193381209

[14] Holick MF . Vitamin D deficiency. N Engl J Med 2007; 357:266–81. 17634462 10.1056/NEJMra070553

[15] Fitzmaurice GM, Laird NM, Ware JH. Applied longitudinal analysis. Hoboken, New Jersey: John Wiley & Sons, 2012.

[16] Harrison SE, Oliver SJ, Kashi DS, et al Influence of vitamin D supplementation by simulated sunlight or oral D3 on respiratory infection during military training. Med Sci Sports Exerc 2021; 53:2555–64. 10.1249/MSS.0000000000002604 PMC8208091 33481482

[17] Laaksi I, Ruohola JP, Mattila V, Auvinen A, Ylikomi T, Pihlajamäki H. Vitamin D supplementation for the prevention of acute respiratory tract infection: a randomized, double-blinded trial among young Finnish men. J Infect Dis 2010; 202:809–14. 20632889 10.1086/654881

[18] Haussler MR, Whitfield GK, Haussler CA, et al The nuclear vitamin D receptor: biological and molecular regulatory properties revealed. J Bone Miner Res 1998; 13:325–49. 9525333 10.1359/jbmr.1998.13.3.325

[19] Ramos-Martínez E, López-Vancell MR, Fernández de Córdova-Aguirre JC, et al Reduction of respiratory infections in asthma patients supplemented with vitamin D is related to increased serum IL-10 and IFNγ levels and cathelicidin expression. Cytokine 2018; 108:239–46. 29402723 10.1016/j.cyto.2018.01.001

[20] He C-S, Fraser WD, Tang J, et al The effect of 14 weeks of vitamin D3 supplementation on antimicrobial peptides and proteins in athletes. J Sports Sci 2016; 34:67–74. 25861808 10.1080/02640414.2015.1033642

[21] Scott JM, Kazman JB, Palmer J, McClung JP, Gaffney-Stomberg E, Gasier HG. Effects of vitamin D supplementation on salivary immune responses during Marine Corps basic training. Scand J Med Sci Sports 2019; 29:1322–30. 31099085 10.1111/sms.13467

[22] Goncalves-Mendes N, Talvas J, Dualé C, et al Impact of vitamin D supplementation on influenza vaccine response and immune functions in deficient elderly persons: a randomized placebo-controlled trial. Front Immunol 2019; 10:65. 30800121 10.3389/fimmu.2019.00065 PMC6375825

[23] Soininen S, Eloranta AM, Schwab U, Lakka TA. Sources of vitamin D and determinants of serum 25-hydroxyvitamin D in Finnish adolescents. Eur J Nutr 2023; 62:1011–25. 36350359 10.1007/s00394-022-03039-y PMC9941269

[24] Engelsen O, Brustad M, Aksnes L, Lund E. Daily duration of vitamin D synthesis in human skin with relation to latitude, total ozone, altitude, ground cover, aerosols and cloud thickness. Photochem Photobiol 2005; 81:1287. 16354110 10.1562/2004-11-19-RN-375

[25] Moriyama M, Hugentobler WJ, Iwasaki A. Annual review of virology seasonality of respiratory viral infections. Annu Rev Virol 2020; 7:83–101. 32196426 10.1146/annurev-virology-012420-022445

